# Dissociation of Size and Distance Effect in Numerical Magnitude Comparison in Less Familiar Number Ranges

**DOI:** 10.5334/joc.486

**Published:** 2026-02-06

**Authors:** Alexis Garsmeur, Roxane Morand, André Knops

**Affiliations:** 1 LaPsyDÉ, CNRS, Université Paris Cité, Paris, France

**Keywords:** approximate number system, discrete symbolic system, numerical cognition, mental number line, interindividual differences, numerosity

## Abstract

The approximate number system (ANS) is thought to mediate symbolic and non-symbolic numerical magnitude comparison. Challenging this view, the dual system model stipulates that non-symbolic comparisons rely on the ANS while symbolic comparisons rely on a discrete semantic system (DSS). In three experiments, the current study tests whether symbolic and non-symbolic magnitude comparisons rely on a common ANS or a DSS by examining the correlation between the size and distance effects in numerical magnitude comparison. We replicated previous studies, which used one-digit numbers 1 to 9, but also aimed to increase variance by using less familiar number ranges. Experiment 1 used a fixed-reference paradigm (reference = 55) with two-digit integers (11–99). Experiments 2 and 3 extended the design to decimals (0.01–0.98) with variable (Experiment 2) or fixed reference (Experiment 3). All experiments additionally included non-symbolic dot comparison in which the expected negative correlation between size and distance effect emerged. Across experiments, size and distance effects in less familiar number ranges were uncorrelated when presented in symbolic format, corroborating the idea that symbolic number comparison relies on a DSS. These findings were moderated by the observation of a significant correlation between size and distance effects in a subsample of participants who showed significant size and distance effects at the individual level. Interpretation of the current results must take into account limitations concerning specificities of multi-digit number processing, the reliability of the effects, and the possible role of unmeasured external factors in shaping the observed correlations.

## Introduction

While our understanding of the neural and cognitive mechanisms underlying the processing of numerical information has advanced over the last few decades, one of the most fundamental questions remains unclear: is numerical magnitude represented in a format-independent manner. One can broadly differentiate non-symbolic formats such as simultaneously presented sets of elements from symbolic formats such as Arabic digits or number words. How numerical symbols, that are initially meaningless, end up representing numerosity is a specific case of the more general “symbol-grounding problem” (Harnad, 1990). In order to manipulate symbols efficiently, children need to connect symbolic representations to their semantic meaning. In the field of numerical cognition, proposals have been made concerning the nature of this connection process, putting forward different cognitive capacities as foundations for symbolic meaning such as subitizing, which is the ability to perceive and keep track of the exact quantities of items in a small cardinality set (Carey, & Barner, 2019) or cognitive control (Leibovich, & Ansari, 2016).

According to one commonly accepted idea, the same semantic code underlies both symbolic and non-symbolic formats (Dehaene, 1992; Piazza et al., 2007). That key behavioral signatures emerge in both formats further substantiates this account. In magnitude comparison tasks, where participants have to indicate the numerically larger of two stimuli, performance improves (faster reaction times, lower error rates) with increasing numerical distance between to-be-compared stimuli. This is referred to as the distance effect. For a given distance, performance decreases with increasing numerical magnitude range which is referred to as size effect. These effects have been reported in experiments using single-digit symbolic number comparison (Moyer and Landauer, 1967), as well as non-symbolic numerosity comparison (Dehaene, 1992). Further evidence from brain imaging research supports this account. The intraparietal sulcus (IPS) is engaged during both symbolic and non-symbolic number processing (Dehaene, Piazza, Pinel, & Cohen, 2003). From these results, it has been inferred that the meaning of symbolic representations (Indo-arabic digits) derives from the activation of a common, analogue psychological mechanism called the approximate number system (ANS). The ANS is therefore supposed to underlie the processing of both symbolic and non-symbolic representations. It is often described by the metaphor of the mental number line, a spatial representation of numbers on a logarithmically organized unidimensional manifold where smaller numbers are located left from larger numbers. Distance and size effects are supposed to be by-products of the ratio effect, a key signature that governs a multitude of sensory dimensions and which can be described by Weber’s law.

However, recent evidence challenges the view of a unitary system, suggesting instead that non-symbolic and symbolic quantities are processed by different cognitive mechanisms (Krajcsi, 2017; Krajcsi & Kojouharova, 2017). In this dual system view, the ANS is limited to processing of non-symbolic quantities. Symbolic numbers that are referring to exact quantity information (as opposed to the approximate nature of the ANS) are processed by the Discrete Semantic System (DSS). The DSS is composed of number representations stored in the form of semantic networks, similar to the mental lexicon (Krajcsi et al., 2016). Numbers are represented by nodes. Nodes are connected via edges that can represent arithmetic relations like parity or whether two numbers are prime numbers or not. The DSS account provides a different explanation for the presence of distance and size effects. According to this account, an increasing frequency of encountering a given symbol decreases the processing time of this piece of information. Because the frequency of encountering a given number symbol and its numerosity is inversely proportional to their numerical magnitude (Dehaene & Mehler, 1992), larger numbers require more processing time compared to smaller numbers (size effect). In a semantic network, the strength of the connections between nodes is the product of their semantic relationship. When two concepts are strongly associated, they are closely linked in the semantic network. This strong connection can lead to competition during retrieval. When accessing any given concept, connected concepts will be co-activated, creating interference and slowing down processing. Therefore, the strength of the link between two number symbols corresponds to their numerical proximity and produces the distance effect.

In order to test this hypothesis, Krajcsi (2017) evaluated the correlation between the distance and size effects for both symbolic and non-symbolic number comparison tasks. According to the unitary ANS explanation, the distance and size effects should be correlated because they emerge from the ratio effect. That is to say, for a given participant, the stronger the distance effect, the stronger the size effect. However, for the DSS account, the distance effect (semantic proximity) and size effect (frequency of exposition) do not emerge from the same mechanism and therefore there is no reason to expect any relationship between the two. Confirming this hypothesis Krajcsi (2017) found a high and significant correlation between distance and size effects when comparing non-symbolic stimuli (dots arrays) but no significant correlation when comparing symbolic stimuli (indo Arabic integers).

However, Krajcsi used numbers from 1 to 9 as stimuli, for which we can speculate that the limited numerical range may mitigate the chances of the size effect to emerge. In the ANS Account, the logarithmic compression is the key feature that leads to a size effect. The limited number range, however, may have impeded the emergence of a size effect. It has been argued, for example, that over the course of an experiment participants acquire automatic stimulus-response mappings that circumvent semantic elaboration when the stimulus set is of limited size (Kunde, Kiesel & Hoffman, 2003).

According to the DSS the size effect emerges from a frequency gradient in the given number range. Since numbers 1 through 9 are the most frequently encountered numbers, the high familiarity may have diminished the variance in the reaction times and hence the gradient may not be sufficiently pronounced to allow for the size effect to emerge. Some authors proposed that the repeated exposure to symbols referring to quantities leads to an association between symbols which gradually decreases the automatic activation of the underlying non-symbolic quantities. This phenomenon is called symbolic estrangement (Lyons, Ansari & Beilock, 2012). In this view, the ANS is a unitary system that only subserves newly acquired stimuli. In consequence, sufficient practice entrains the processing of symbols via another system that could potentially correspond to the DSS described by Krajcsi (2017).

One way to test this idea is to evaluate the correlation between the distance and size effects for a wider range of numbers. Therefore, in the first experiment, we applied the paradigm used by Krajcsi (2017) to two-digit integers ranging from 11 to 99. We expected that this range would give us sufficient variation to observe size effects. Additionally, this allowed us to directly match symbolic and non-symbolic quantities without applying a multiplicative transformation to the symbolic quantities when creating the non-symbolic numerosities as in Krajcsi (2017).

For the DSS account, size effects are not expected because the range of exposure frequency in the number range 11 to 99 is smaller compared to the range between one and nine (Pajot et al., 2025). Accordingly, similarly to the one-digit integers, there should be no correlation between size and distance. On the other hand, the symbolic estrangement hypothesis account predicts that with sufficient variation a correlation should emerge for less familiar stimuli. However, this correlation could be weaker than with the non-symbolic quantities depending on the degree of familiarity and therefore “estrangement” with these symbols.

## Experiment 1

To probe any potential correlation between the distance and size, Experiment 1 was designed as a magnitude comparison task where symbolic and non-symbolic magnitudes between 11 and 99 were compared with a fixed reference (55). This increased the size range (calculated as the sum of the two to-be-compared numbers) which varies between 66 and 154 (excluding 110) in the current task, compared to Krajcsi (2017), where sizes ranged from 3 to 17. We reasoned that this design change would allow for more variability in RT and therefore increase the chance of a potential correlation to emerge.

### Participants

We recruited 34 participants through advertisements on college campuses. Most participants were students in the Master “Economie et Psychologie” of Paris 1 Panthéon Sorbonne, all residing in France. At the beginning of the experiment, participants were asked to provide personal information, including their age, gender, and handedness. We excluded one participant from the analysis because his error rate was superior to 3 SD above the mean error rate of the group. The final sample included 14 males and 18 females (one participant did not provide this information). The mean age was 26.45 years (ranging from 20 to 59). The protocol was carried out in compliance with the ethical standards of the Declaration of Helsinki.

### Stimuli and Materials

We used MATLAB to generate stimuli for both formats.

#### Non-symbolic quantities (sets of dots)

Numerosities ranged from 11 to 99 and each numerosity (except 55) was presented twice, counterbalancing the presentation side of each number. Thus, 88 trials were shown twice.

To control for the non-numerical features that inevitably covary with numerosity, we generated eight stimulus sets: In a given set, either the total surface (TS) area or convex hull (CH) was kept constant between the two presented dots arrays. The visual property that obligatorily varied between the two presented arrays was either congruent or incongruent with the numerosity, yielding 4 stimulus sets. Since the larger stimulus was equally often presented on the left or right side from central fixation, we created these four sets for each side. For numbers before the reference, size increase as the distance to the reference decrease. For numbers after the reference, size increase as the distance to the reference increase. Therefore, in the stimuli set distance and size are uncorrelated.

#### Symbolic quantities (two-digit numbers)

Analog to the non-symbolic condition, we presented the numbers 11 through 99 (except 55). Each of the 88 numbers was shown twice.

#### Design and Procedure

To mitigate the potential influence of the primacy effect, the order of the formats alternated between participants. The experiment was controlled by Psychtoolbox (Brainard, 1997), running on MATLAB R2023b. The following instructions were presented to the participants with symbolic stimuli: “Please decide whether the shown number is larger or smaller than 55! Press g when it is smaller. Press h when it is larger.” With non-symbolic stimuli, participants were instructed as follows: “Please indicate which side the more numerous set is displayed on! Press g for left and h for right!”. When participants took too much time to respond, a message appeared on the screen: “Please respond as fast and as accurately as possible.” Ten practice trials preceded each format.

#### Non-symbolic quantities

In the numerosity comparison, two sets of dots were presented to the left side and right side of the screen center, respectively. One of the two sets had a numerosity of 55 dots (reference) while the other changed numerosity across trials. For each numerosity, the reference was displayed on the left in 50% of the trials. Dot arrays were presented until participants responded or a maximum response duration of 2000 ms. Response was followed by an empty screen of 500 ms until the next trial. The 176 trials were presented in 4 blocks, each lasting for approximately 2 minutes. In order to control for confounding, 25% of stimuli were incongruent in Total area (the largest number also had the largest Total area) and 25% of the stimuli were congruent in the Convex hull (the largest number also had the largest Convex hull). The 50% remaining trials were congruent in both visual properties.

#### Symbolic quantities

In the symbolic magnitude comparison task, participants were instructed to compare a number displayed in the center of the screen to the number 55. The displayed number remained visible until the participant pressed a key, or a maximum response duration of 2000 ms. After the response, a blank screen was shown for 500 ms before the next trial started. The experiment consisted of 4 blocks, each lasting approximately 2 minutes.

### Analysis

We used R (Team, 2010) for all analyses.

First, we excluded trials with erroneous, missed or premature (reaction times (RTs) < 200 ms) responses. Next, we excluded trials that fell outside a participant- and experiment-wise range of mean RT ± 3 standard deviations (SDs). Finally, we excluded participants if their error rate (or mean reaction time) in at least one of the two tasks exceeded the mean group error rate (or mean reaction time) by more than 3 SDs. Applying this criterion we excluded one participant. All analyses are based on the data from the remaining 33 participants.

To capture the distance effect, we calculated the absolute distance between the probe number and the reference, following Krajcsi (2017). The size effect was assessed as the sum of the reference and the probe number (55 + displayed number).

To assess the presence of a distance and size effect at the group level, we computed a 2 x 2 repeated measures ANOVA with size and distance as factors using the ezANOVA package of RStudio. We categorized distances 1 to 22 as small distances and the distances 23 to 44 as large distances. Number pairs with sums between 66 (55 + 11) and 109 (55 + 54) were categorized as small sizes and pairs with sums between 111 (55 + 56) and 154 (55 + 99) as large sizes. ANOVA’s results are reported in supplementary materials. To exploit the continuous nature of the stimuli, we also tested distance and size effects at the group level using a linear mixed model with participants as random intercepts.

In order to assess the presence of a ratio effect in both format condition, we tested the ratio effect at the group level using another linear mixed model with random intercept for participants. Ratio for a given pair was calculated as the smallest number between the probe number and the reference divided by the largest number between the two.

We also calculated the slopes of the distance effect for numbers below 55 and those above, for each participant and each format conditions (symbolic and non-symbolic). Under the assumption of a logarithmic compression of the number representation, we would expect the slopes for numbers above 55 to be less steep compared to numbers below 55. In order to compare the magnitude and not the directionality of slopes, we ran a dependent sample t-test on the absolute values of slopes of the linear regression of presented numbers on RTs, separately for each format. Moreover, we conducted an analysis on the mean response time across participants for symbolic/non-symbolic numbers below and above 55. We expected to see in both formats a larger response time for symbolic/non-symbolic numbers above 55 than for symbolic/non-symbolic numbers below 55 (as the mean ratio is supposed to be closer to one for the set of numbers above 55).

For each participant and each format, we assessed size and distance effects via unstandardized regression coefficients in a multiple stepwise regression with distance as the first and size as the second regressor. Size was introduced second because we used a fixed reference comparison task in which the distance effect counteracts the size effect for numbers higher than the reference.

Unstandardized regression coefficients of the distance and size effects were calculated for all participants for both formats. Regressions between distance and size effects coefficients were first calculated using (a) all participants, then recalculated (b) excluding outliers’ coefficients with a two-dimensional outlier detection using an ellipse with a radius set as 95% confidence interval, multivariate t-distribution were assumed (Fox and Weisberg 2011; Friendly and Monette 2013) and finally (c) using only participants having both a significant size and distance effects (Roth et al., 2024). The analysis after excluding participants according to (b) and (c) aimed at restricting subsequent correlation analyses to those participants who do manifest both effects at the individual level. It can be considered a conservative way of identifying those who rely on the ANS. Note that it does not – in and by itself – predict that there is a significant correlation between these effects, however.

The Pearson’s product correlation between distance effects coefficients and size effects coefficients across participants was calculated for both formats. Since the results of the regression analyses without outliers and with both significant size and distance effects produced effectively identical results as the one including all participants (n = 33), they will not be reported here.

In order to test the presence of a common mechanism across both format we develop a second measure that was not present in Krajcsi (2017). We computed the ratio effect directly for both format conditions and then correlated the two effects among the two tasks. If the same system characterizes by the ratio effect underly symbolic and non-symbolic performances we should observe a high correlation between both ratio effects. To calculate the ratio effect, we regressed RT of given stimulus on its ratio using a simple linear model.

Finally, we computed the split-half reliability and corrected, using the Spearman-Brown correction, our correlation coefficients for the lack of reliability of our variables following the method described in Krajcsi (2017). Negative correlation scores were considered as equivalent to a reliability score of zero. Conversely, corrected correlation coefficient superior to 1 (or –1), where considered equivalent to a perfect correlation of 1 (or –1). This allowed us to correct for the presence of noise in, for example, the visual perception of our stimuli that could differ from one task to another (symbolic vs non-symbolic). Different levels of noise could decrease the strength of the correlation between our two effects for a given task, possibly creating artificial differences of correlation coefficients between tasks.

### Results

Accuracy differed between the symbolic and non-symbolic format (mean = 95.21%, SD = 2.60 and mean = 63.22%, SD = 4.03, respectively), with notably higher variability observed in the dot condition. This variation originates from the pairs with ratios close to 1, such as 54–55, 53–55, or 52–55, which were below detectability threshold which is around 1.1 for adults (Halberda, Mazzocco, & Feigenson, 2008; Pica et al., 2004).

Non-symbolic comparisons (*t*(32) = 8.72, *p* < .001, *d* = 1.46) were slower (Mean (SD) = 777 (167) ms) compared to symbolic two-digits integers (Mean (SD) = 562 (68) ms).

For non-symbolic quantities, a linear mixed model at the group level on all participants’ trials with participant as random intercepts yielded significant effects for distance (*t*(3586) = 3.104, *p* = .002), size (*t*(3586) = 3.307, *p* < .001) but no significant effect for their interaction (*t*(3586) = 1.162, *p* = .245). The second linear mixed model with ratio as the only regressor also yields a significant effect (t(3588) = 22.16, p < .001). For symbolic numerosities, a linear mixed model showed a significant effect for distance (*t*(5388) = 6.175, *p* < .001), size (*t*(5388) = 2.689, *p* = .007) and their interaction (*t*(5388) = 2.512, *p* = .012). As for non-symbolic task, the second linear mixed model with ratio as the only regressor also yields a significant effect (t(5390) = 13.87, p < .001).

If size and distance effect reflect a common underlying representation governed by Weber’s law, we should see that overall reaction times and the distance effect are modulated by the overall magnitude range in which these markers are computed. In line with this, participants reacted significantly faster to numbers below 55 (M = 743 ms, SD = 149 ms) compared to numbers above 55 (M = 815 ms, SD = 187 ms, *t*(32) = 7.47, *p* < .001, *d* = 0.32), for non-symbolic quantities. Conversely, for symbolic stimuli, participants reacted faster to numbers above 55 (M = 556 ms, SD = 68 ms) than to numbers below 55 (M = 568 ms, SD = 70 ms, *t*(32) = 2.36, *p* = .025, *d* = 0.16).

**Figure 1:**
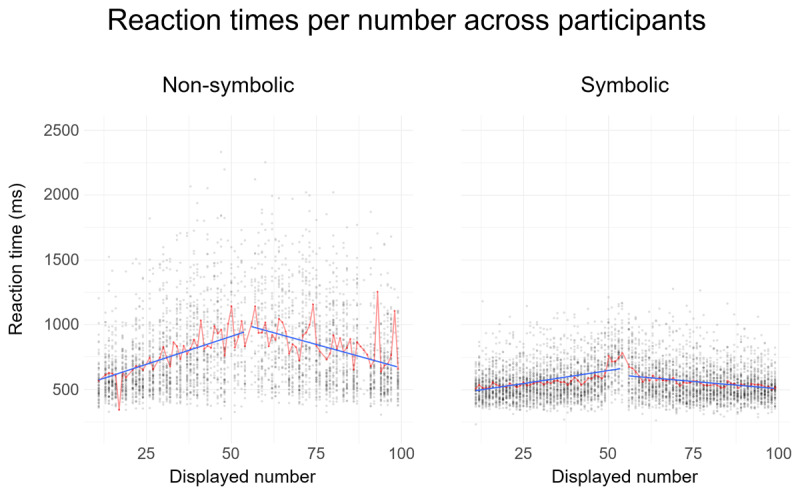
Reaction times plotted as a function of the displayed numbers in the non-symbolic (left) and symbolic (right) conditions in Experiment 1. Blue line represents the regression of numerical distance on reaction time, separately for both formats and numbers larger or smaller than the reference (55). Red line represents the mean reaction time for each presented number.

At the group level, the distance effect for non-symbolic quantities, as measured via the magnitude (absolute value) of the slope of the regression of number on RT, was larger for numbers below 55 (M = 8.79 ms/number, SD = 4.93 ms) compared to quantities above 55 (M = 7.03 ms/number, SD = 4.24 ms, *t*(32) = 2.52, *p* = .017, *d* = 0.38). Similarly, for symbolic stimuli, the distance effect was more pronounced for numbers below 55 (M = 3.57 ms/number, SD = 1.46 ms) compared to numbers above 55 (M = 2.32 ms/number, SD = 1.58 ms, *t*(32) = 3.52, *p* = .0013, *d* = 0.80) (see Figure 1). This could reflect the stipulated compression of the underlying magnitude representation in both formats.

**Figure 2:**
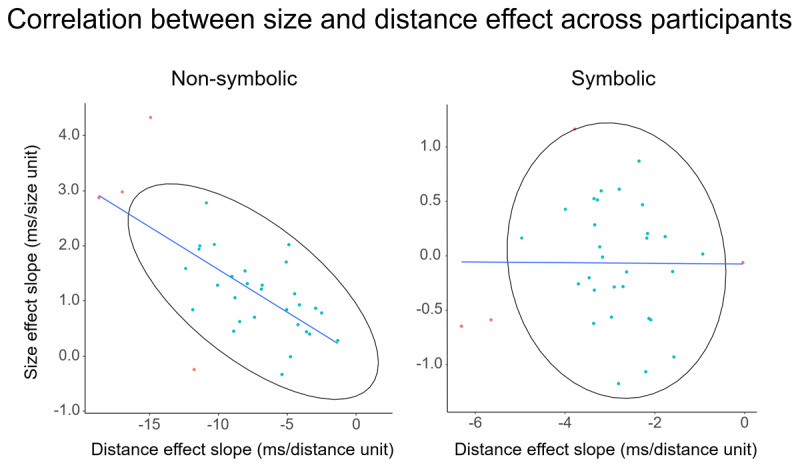
Relation between the distance and size effect unstandardized regression coefficients, displayed on scatterplots and measured with correlation coefficients for dot comparison.

To probe the notion that distance and size effects emerge from a unitary system (ANS account), we computed the correlation of the unstandardized regression coefficients of the two effects in the two formats (see Figure 2). The estimated correlation coefficient for the non-symbolic task was *r*(31) = –.65 (p < .001), implying a very high degree of correspondence which is in line with the idea that both effects emerge from the ratio effect. For the symbolic task the estimated correlation coefficient was not significant (*r*(31) = –.01, *p* = .968). These results closely replicate the findings by Krajcsi (2017) who found correlations of *r* = –.88 and *r* = –.96 for the non-symbolic task and *r* = –.11 and *r* = –.13 with symbolic one-digit integers. Confidence intervals of correlation coefficients didn’t overlap between non-symbolic (95% CI[–.86, –.44]) and symbolic tasks (95% CI[–.37, .35]). The correlation between the ratio effects of both formats was *r*(31) = 0.01 (p = .962).

**Figure 3:**
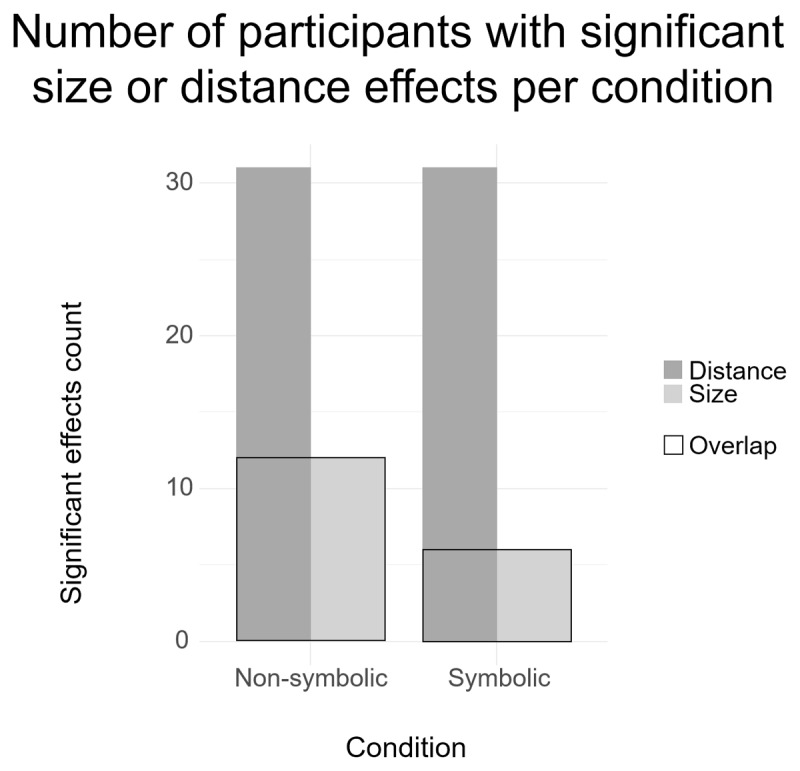
Histogram of participants with significant size or distance effects in Experiment 1 in two digits symbolic (Symbolic) and the dot arrays (Non-symbolic) conditions. The black rectangles (overlap) depict the number of participants showing both effects in both formats.

Recently, the idea that interindividual variability should not be considered as noise but reflect differences in cognitive processes has gathered some interest (Roth et al., 2024). Roth and colleagues investigated the significance of the SNARC effect at the individual level and argued that this effect, albeit robust at the group level, is not present in all participants. This discussion has inspired us to rerun the above analyses with participants showing both significant distance and size effects at the individual level only. In the non-symbolic task, most distance effects (31 participants; 93.94% of the sample) and a small portion of size effects (11 participants; 33.33% of the sample) were significant (see Figure 3). For symbolic two-digits integers, most of the distance effects were significant (31 participants; 93.94% of the sample) but the number of significant size effects was greatly reduced (6 participants; 18.18%). Therefore, in the case of the symbolic two-digit integers we were not able to run a meaningful regression analysis on so few participants. Concerning the non-symbolic task, results remained unchanged, the correlation between significant size and distance effects was still significant.

The split half reliability analysis correction increased the strength of our correlation in both formats (Non-symbolic: *r* = –.65; *r* corrected = –.88; Symbolic two-digit integers: *r* = –.01; *r* corrected = –.18). Complete report of reliability scores for each condition and experiment can be found in supplementary materials.

### Discussion

Experiment 1 replicated main results obtained by Krajcsi (2017). We observed a clear correlation between size and distance effect in the non-symbolic format, corroborating existing evidence that the processing of non-symbolic numerosities relies on a unitary analog magnitude representation that is governed by Weber’s law (Dehaene, 2003). For symbolic stimuli, we observed no correlation between size and distance effects, undermining the idea that symbolic number processing is governed by the same system as non-symbolic quantity processing. This result may be due to the incoherent emergence of a size effect in the symbolic format, as we hypothesized for the results by Krajcsi (2017).

The absence of a coherent size effect in all analyses could be due to the overlearned nature of integers. We hypothesized that using less familiar and automated symbolic stimuli should produce behavioral signatures typical of the approximate magnitude system. In experiment 2, we therefore used decimals ranging from 0.01 to 0.98.

## Experiment 2

For experiment 1, we reasoned that using a larger stimulus range would increase the chances to observe a size effect and thereby a correlation between size and distance effects that would follow from accessing an approximate number representation that is governed by Weber’s law. However, even when using two-digit integers, we failed to observe a correlation between size and distance effect. One may argue that two-digit numbers are still too familiar and overlearned and therefore mostly governed by a discrete semantic system. To test this hypothesis, we used less familiar and less automatized stimuli in Experiment 2: decimal numbers in the range 0.01 to 0.98.

### Participants

We recruited 47 participants via an online platform (https://www.risc.cnrs.fr/), residing in greater Paris region. Participants were compensated with 10 euros for a test duration of one hour. Testing was realized in the sound attenuated testing booth at the LaPsyDÉ laboratory. We excluded three participants from the analysis because their error rate of at least one of the three conditions was superior to three standard deviations above the mean error rate of the entire sample for the given condition. The final sample included 27 females and 17 males, with an age range of 18–75 and a mean age of 45.7 years. The experiment was carried out in compliance with the ethical standards of the Declaration of Helsinki and was approved by the local ethics committee (2023-96-KNOPS-GARSMEUR).

### Materials

In experiment 2, we probed the common involvement of the ANS in both symbolic and non-symbolic quantity comparison with less familiar symbolic stimuli, that is decimals. Experiment 2 comprised three parts: a replication of Krajcsi (2017) using single-digit numbers between 1 and 9, a replication of Krajcsi (2017) using non-symbolic quantities, and a comparison of two decimals ranging from 0.01 to 0.98. Symbolic stimuli sets were generated using Python 3.11. Note that we also created and tested negative decimals ranging from –0.98 to –0.01. The results of this number range will be reported elsewhere. Non-symbolic stimuli were generated using MATLAB.

#### Non-symbolic quantities

The non-symbolic stimuli were created following the procedure described in Krajcsi (2017): We generated all possible pairs with numbers between 1 and 9 (except for same number pairs) and multiplied each number by five to prevent stimuli in the subitizing range (1–4). In order to increase the reliance on numerical information of the dot arrays (rather than non-numerical features). Going beyond previous studies, we aimed at controlling for the influence of non-numerical stimulus features by creating two sets of non-symbolic stimuli (sets of dots). The first set controlled for the item size (and the density) of the two to-be-compared dot arrays. The second set controlled for the convex hull (and the overall occupied area). For each of the 36 possible pairs of integers in the two sets (item size control or convex hull control), we had eight stimuli, yielding a total of 36 × 8 × 2 = 576 trials per participant.

#### Symbolic integers task

Following the procedure in Krajcsi (2017), we generated all possible pairs of numbers between 1 and 9, excluding identical number pairs. Each number pair was presented eight times to each participant, leading to a total of 288 symbolic integers trials per participant.

#### Symbolic decimal task

In order to extend Krajcsi (2017) results to stimuli that were less familiar to participants compared to the one-digit numbers in Krajcsi (2017), we used decimals with two decimal positions after the leading zero: First decimal position (DP_1_) after the leading zero and second decimal position (DP_2_) after the leading zero. Since the frequency of numbers in our environment follows Benford’s law, two-digit numbers are less frequent than single digit numbers (Dehaene & Mehler, 1992). Decimals with leading zeros (e.g. 0.52) have not been included in frequency analyses. Hence, we speculated that they should be less commonly encountered than natural numbers. In real life, we rarely encounter integers written with a leading zero (e.g. “04” compared to “4”). Using decimals therefore allows us to have the same number of digits with every number in the entire range from 0.01 to 0.99 written in a conventional way (e.g. 0.04 vs. 0.12 is less counterintuitive than 04 vs. 12).

Generation of number pairs was pseudo-randomized with the following restrictions. We excluded number pairs with the same DP_2_ digit (e.g. 0.35 vs 0.45) or pairs including tie numbers (e.g. 0.77 vs 0.72). The symbolic extension task comprises a total of 338 number comparison pairs.

For non-identical DP_2_ trials, we aimed to stay as close as possible to the identical DP_1_ stimuli to facilitate comparison between the two tasks. When non-identical DP_1_ trials share the same DP_2_ digit, the numerical distance between the two numbers is 10 units (e.g. 65 vs 75). However, the maximum possible distance in identical DP_1_ trials is 9 units. Introducing distances of 10 units or more in identical DP_1_ trials would effectively convert them into non-identical DP_1_ trials.

With two-digit decimals, participants could, in theory, focus only on the DP_1_ when deciding on the larger of two decimals. Even though previous studies have demonstrated that participants cannot totally ignore irrelevant information within two-digit numerals (Nuerk et al., 2001), we introduced decimals with identical DP_1_ (e.g. 0.42 vs. 0.48) to increase the salience of DP_2_ and thereby oblige participants to integrate both digits in a given numeral.

##### Identical DP1 comparisons

For comparisons with zero and nine in DP_1_, the exclusion of tie numbers prohibited including pairs with a distance of 0.09 (e.g. 0.00 vs 0.09 and 0.90 vs 0.99).

For trials with identical DP_1_, we generated numbers using the following method: For each DP_1_ digit between 0 and 9 and for each distance between 1 and 9, we generate two random numbers that have the given DP_1_ number, and that are separated by the given absolute numerical distance. By doing so, our stimuli set is not biased in its sampling of distances, the distribution of trials by distance is homogeneous (except for the DP_2_ distance 9, which has less trials than the other due to the lack of DP_1_ 0 or 9). On the other hand, the distribution of trials by number presented is biased: certain distances exclude certain numbers (e.g. the DP_2_ distance 8 cannot be represented by numbers pairs where one DP_2_ digit is 5).

For each DP_1_ and DP_2_ distance we generated 2 trials (except for DP_2_ distance 9 and DP_1_ 0 and 9). Thus, we obtain a total of: 8 × (2 × 9) + 2 × (2 × 8) = 176 trials.

##### Comparisons with different DP1s

Stimulus pairs for comparisons with non-identical DP_1_ digits were generated the same way, except that for a given DP_1_ digit between 0 and 9, our number pairs were constructed by comparing a given number n_1_ with a_1_ and b_1_ as DP_1_ and DP_2_, with a number computed as n_2_ = a_1_ ± 1 and b_1_ ≠ b_2_.

Thus, we had 2 × (2 × 9) = 36 trials per DP_1_ digit (2 × 9 = 18 trials with the upper DP_1_ and 18 trials with the inferior DP_1_). The 0 (9) DP_1_ digits had only 18 comparisons, because no DP_1_ digit is smaller (larger) than DP_1_ 0 (9). In total, we obtained 9 × (2 × 9) = 162 trials for non-identical DP_1_ conditions.

Compared to Krajcsi (2017), we increased the size range (defined as the sum of n_1_ and n_2_) of the symbolic decimals stimuli, ranging from 0.03 (i.e. 0.01 + 0.02) to 1.95 (i.e. 0.98 + 0.97). This is an important prerequisite for a linear regression with size as one of its predicting variables (size effect).

### Design and Procedures

We went from a fixed reference comparison task in the first study to a two-number comparison tasks in the second study because, in order to prevent compatibility effects and to have both sufficient variation in size and sufficient trials by distances, it wasn’t possible to have the same reference number across the 9 DP_1_.

Two numbers were presented to the participants, respectively to the left and right of the fixation cross presented in the center. Their task was to indicate the larger of the two numbers by pressing the corresponding response button (“s” for left and “k” for right) as quickly as possible while making as few mistakes as possible. Instructions were written in French.

Each participant effectuated three different tasks. Order of tasks was identical for all participants, starting with the non-symbolic numerical magnitude comparison task (dots arrays), followed by one-digit integers, and finally symbolic decimals. The two first formats were replications of Krajcsi (2017). The symbolic decimal condition comprised two sub-conditions: pairs with identical DP_1_ digits, followed by pairs with non-identical DP_1_. Each condition and sub-condition started after completing a practice block with feedbacks to make sure that participants had understood the task.

A trial started with the presentation of a fixation cross for 300 ms, followed by an empty screen for 300 ms. Then, two numbers were presented until the keypress or a maximum of 2500 ms. A response was followed by an empty screen for 300 ms before the next trial started.

### Analysis

All statistical analyses were done using R.

Applying the same outlier conditions as in Experiment 1, three outlier participants were excluded. All analyses are based on the data from 44 participants. At the trial-level, the same criteria as in Experiment 1 led to the exclusion of 2212 (8%) of non-symbolic trials, 1570 (11%) of symbolic integers trials and 5891 (17%) of symbolic decimals trials.

For each participant, outlier response latencies were defined as being outside the individual mean response latency per condition plus/minus 3 SDs or below 200 ms after stimulus onset. Reaction time analyses were conducted on the response set after excluding practice trials (presented at the beginning of each task), outliers (as defined above), and missed trials. Error analyses were conducted on the response set with only the practice trials excluded.

In order to simplify the understanding of the results, distance and size were calculated on the numbers multiplied by 100. This linear transformation does not affect the statistical analysis. The distance of a number pair (n_1_–n_2_) was calculated as the absolute difference between the two numbers being compared (abs(n_1_–n_1_)). Size values were calculated by taking the sum of the two numbers inside a pair (n_1_+n_2_).

Comparable to the first experiment, we tested whether the distance effect varied as a function of size or distance by computing a 2 × 2 repeated measures ANOVA (Distance: Small/Large × Size: Small/Large) using the ezANOVA function of the ‘ez’ package. For the non-symbolic tasks, we applied the same classification method between small and large distances and sizes as in the first experiment. For Symbolic integers, we classified distances ranging from 1 to 4 as small distances and distances ranging from 6 to 9 as large distances. Sizes (i.e. sum of the to-be-compared numbers) ranging from 3 to 9 were considered as small; sizes ranging from 11 to 17 as large. In order to have a sufficient number of trials in each design cell of the decimal condition, we separated distances and sizes (defined as the sum of the DP_1_ of the two given numerals) in two bins. The small distances bin comprised distances 1, 2, 3 and 4, while the large distances bin comprised distances 6, 7, 8 and 9. Same for sizes, sum of DP_1_ : 0, 1, 2, 3, 4, 5, 6, 7, 8 were aggregated together inside the Small sizes bin, while sum of DP_1_ : 10, 11, 12, 13, 14, 15, 16, 17 and 18 constituted the Large sizes bin. The results adopting a classical ANOVA approach are reported in supplementary materials.

In order to assess the presence of distance and size effects at the group level, we also ran a linear mix model analysis on all trials of all participants, with size and distance as regressors and random intercepts for each participant.

We assessed the presence of a ratio effect in both format condition by directly testing the ratio effect at the group level using a separate linear mixed model with random intercept for participants.

At the individual level, the linear regression slopes of the distance and size effects were calculated for all participants for all conditions on all trials in a given condition. Contrarily to the first experiment, we did not run a stepwise linear regression to evaluate effects because the design of the second experiment was done in order to make distance and size orthogonal to each other. Two linear regressions, the first with distance and the second with size as regressors, were computed to estimate distance and size effects slopes for each participant. The correlation between distance and size across participants were first calculated using (a) all participants, then recalculated (b) excluding outliers’ slopes with a two-dimensional outlier detection using an ellipse and finally (c) using only participants having both a significant size and distance effects.

The regression without outliers produced the same results as the one including all participants (n = 44) and will therefore not be reported here. The Pearson’s product correlation between distance effects slopes and size effects slopes across participants was calculated for all conditions.

Following the first experiment, we calculated the Pearson’s product correlation between distance effects slopes and size effects slopes across participants for all conditions, and then performed a split half reliability analysis.

Similar to the first experiment, we computed the ratio effect for all conditions and then correlated the two effects among the two tasks.

### Results

Accuracy differed between the non-symbolic quantities (Mean = 95.55%, SD = 1.63), symbolic integers (Mean = 97.66%, SD = 2.20) and symbolic decimals (Mean = 95.64%, SD = 3.13).

Compared to symbolic integers (Mean (SD) = 573(125) ms), non-symbolic comparisons (Mean (SD) = 704(171) ms) were significantly slower (*t*(43) = 9.206, *p* < .001, *d* = 0.779). Symbolic decimals comparisons (Mean (SD) = 890(173) ms) were significantly slower (*t*(43) = 11.003, *p* < .001, *d* = 1.078) than non-symbolic comparisons. Symbolic decimals comparisons were significantly slower (*t*(43) = 25.798, *p* < .001, *d* = 1.741) than symbolic integers comparisons. Importantly, standard deviation comparison between symbolic decimals (Mean_SD (SD_SD) = 195(47) ms) and symbolic integers (Mean_SD (SD_SD) = 114(43) ms) showed that symbolic decimals RT were significantly more dispersed (t(43) = 16.616, p < .001, *d* = 1.777). Therefore, we elicited a greater variability in symbolic decimals compared to symbolic integers, increasing our chance of observing a significant correlation if there is one.

In the LMM analysis on all trials with participants as random intercepts, all distance, size and ratio effects were significant in the three main conditions (Non-symbolic, Symbolic integer, Symbolic decimal; see Table 1). Interaction between distance and size were significant only in non-symbolic and symbolic integer conditions.

**Table 1:** The unstandardized regression coefficients, t and p values for the distance, size, distance-size interaction and ratio effects of the three experimental conditions (non-symbolic, symbolic integer, and symbolic decimal). Distance, size and their interaction effects were evaluated within the same linear mixed model. To evaluate the ratio effect, we employed an additional linear mixed-effects model. In all models, participants were treated as random intercepts to account for individual variability. Degrees of freedom of Distance, Size and their interaction for the non-symbolic, symbolic integer and symbolic decimals are respectively 2.528e+04, 1.286e+04 and 2.940e+04. Degrees of freedom of Ratio for the non-symbolic, symbolic integer and symbolic decimals are respectively 2.528e+04, 1.286e+04 and 2.941e+04.


FORMAT	DISTANCE EFFECT			SIZE EFFECT			SIZE*DISTANCE EFFECT			RATIO EFFECT		
			
	*coeff*	*t*	*p*	*coeff*	*t*	*p*	*coeff*	*t*	*p*	*coeff*	*t*	*p*

Non-Symbolic	–9.861e–03	17.257	<.001	3.957e–03	26.313	<.001	2.261e–05	2.007	.045	5.460e–01	98.94	<.001

Symbolic Integers	–2.672e–02	11.543	<.001	3.079e–03	5.243	<.001	5.782e–04	2.558	.011	1.941e–01	44.10	<.001

Symbolic Decimals	–5.123e–03	5.302	<.001	1.663e–02	3.481	<.001	1.158e–03	1.343	.179	6.756e–02	9.408	<.001


#### Do size and distance effects correlate?

In the non-symbolic task, size and distance effects were highly correlated (*r*(42) = –.81, p < .001), in line with the idea of a unitary dependence on ratio.

For the symbolic integer task, however, the estimated correlation coefficient was not significant (*r*(42) = –.19; *p* = .227). These results closely replicate the findings by Krajcsi (2017) who found a correlation of *r* = –.88 and *r* = –.96 for the non-symbolic format and *r* = –.11 and *r* = –.13 for the symbolic format.

The differences between the correlations in the non-symbolic and symbolic integer tasks were significant in both Krajcsi’s study and ours. When jointly analyzing all symbolic decimals conditions, we observed a significant correlation between size and distance effects (*r*(42) = .40; *p* = 0.007), contradicting Krajcsi’s findings (2017) with less familiar decimals (see Table 2).

**Table 2:** Correlation coefficients of distance and size effect, separately for each format. Significant correlations are shown in bold print.


	CORRELATION WITH OUTLIERS	CORRELATION WITHOUT OUTLIERS

Non-Symbolic	**–.81** (95% CI[–.92, –.70])	**–.88** (95% CI[–.95, –.81])

Symbolic Integer	–.19 (95% CI[–.49, .11])	.01 (95% CI[–.30, .32])

Symbolic Decimal	**.40** (95% CI[.14, .66])	**.36** (95% CI[.09, .63])

Symbolic Decimal identical DP_1_	.11 (95% CI[–.19, .41])	.11 (95% CI[–.19, .41])

Symbolic Decimal non-identical DP_1_	**.44** (95% CI[.19, .69])	**.39** (95% CI[.13, .65])


There was no overlap between non-symbolic and Symbolic conditions (with and without outliers) correlation coefficients confidence intervals. The focus of experiment 2 was to explore the correlation of size and distance effect in less familiar numbers such as decimals. In contrast with previous studies, we observed a significant correlation between size and distance effects with decimals. Since the literature on decimals is scarcer, we explored in more detail participants’ performance in these tasks.

A two-sided t-test between decimals with identical DP_1_ (mean(SD) = 779(163) ms) and with non-identical DP_1_ (mean(SD) = 931(172) ms) showed that reaction times were higher for pairs with non-identical DP_1_ (*t*(43) = 16.17, *p* < .001, *d* = 0.89).

For the decimals with identical DP_1_ stimuli, we obtained a regular main effect for distance (*F*(1, 43) = 227.77, *p* < .001, *η*_p_^2^ = .84) and the size of DP_1_ (*F*(1, 43) = 19.344, p < .001, *η*_p_^2^ = .31). However, the interaction between distance and DP_1_ size was not significant (*F*(1, 43) = 1.601, *p* = .213, *η*_p_^2^ = .036).

For decimals with non-identical DP_1_, we observed a reversed main effect of distance (*F*(1,43) = 11.616, *p* = .0014, *η*_p_^2^ = .21) and size of DP_1_ (*F*(1,43) = 13.761, *p* < .001, *η*_p_^2^ = .24) but no interaction (*F*(1,43) = 0.174, *p* = 0.679, *η*_p_^2^ = .004).

Contrary to Krajcsi (2017), we observed a significant correlation of size and distance effect for decimals (*r* = .40; *p* = .007). To understand the origin of this correlation, we separately analyzed comparisons with identical and non-identical DP_1_ digits. We found that the overall significant correlation of size and distance effect in decimals was driven by the significant correlation of non-identical DP_1_ pairs (*r* = .44; *p* = .003) whereas no significant correlation was observed for identical DP_1_ number pairs (*r* = .11; *p* = .687).

It is important to note that in the case of non-identical DP_1_ the correlation coefficients were positive because the distance effect was positive (see Figure 5), contrarily to the identical DP_1_ and more generally to the well-established results in the field of mathematical cognition demonstrating a negative relationship between the numerical distance of two compared numbers and the reaction time. We believe that this result is due to the perfect negative correlation of DP_1_ and DP_2_ that resulted from the constraints that the absolute distance between two numbers was set to be ≤ 9 and the DP_1_ distance was set to 0.1. For example, in the decimal pair 0.48 vs. 0.56, the absolute distance (|0.56 – 0.48|) is 0.08, while the absolute DP_2_ distance (|0.06 – 0.08|) is 0.02. For a pair such as 0.49 vs. 0.51, the absolute DP_2_ distance is 0.08 while the absolute distance between these numbers is 0.02. These examples demonstrate that decreasing the absolute distance increases DP_2_ distance. We hence believe that the distance effect in our set reflects the comparison of DP_2_ digits. Since DP_2_ digit distance is both negatively correlated to reaction time and absolute distance, it creates a positive relationship between absolute distance and reaction times. Therefore, if we calculate distance effects based on DP_2_ distances, the correlation between distance and size effects among positive non-identical DP_1_ pairs becomes negative (*r* = –.44; *p* = .003).

The ANOVA of non-identical DP_1_ suggests a significant size effect. But the fact that this main effect is not qualified by a significant interaction with distance underscores our interpretation that the size effect reflects a distance effect among DP_1_ digits rather than a canonical size effect. Therefore, the presence of a correlation between distance effects and “size” effects in our non-identical DP_1_ stimuli set could be the result of a correlation between the distance effect of the DP_1_ and the distance effect of the DP_2_. Taken together, it seems that we failed to observe a proper size effect for symbolic decimals. This may be due to the presentation mode where the simultaneous presentation of two decimals allows for separately comparing DP_1_ and DP_2_ digits of both numbers instead of comparing their overall numerical magnitude. The reverse distance effect in non-identical DP_1_ pairs provides further credence to this reasoning. These results conceptually replicate previous reports of a reverse distance effect for two digits number pair comparisons (Brysbaert, 1995) and more recently for decimal numbers (Rosenberg-Lee et al., 2023).

Correlation between ratio coefficients across tasks showed a significant correlation between ratios of non-symbolic and symbolic integer conditions and between symbolic conditions themselves (see Table 3). We also computed the correlation between the ratio effects of both sub-conditions of the decimal fraction task (*r*(42) = 0.47, p = .001).

**Table 3:** Correlation coefficients of ratio effects, between each conditions (and sub-conditions inside the Symbolic Decimal). Significant correlations are shown in bold print.


	SYMBOLIC INTEGER	SYMBOLIC DECIMAL	SYMBOLIC DECIMAL IDENTICAL DP_1_	SYMBOLIC DECIMAL NON-IDENTICAL DP_1_

Non-Symbolic	**.56**	–.19	–.11	–.26

Symbolic Integer		**–.43**	**–.36**	**–.50**


#### Are distance and size effects ubiquitous in the sample?

**Figure 4:**
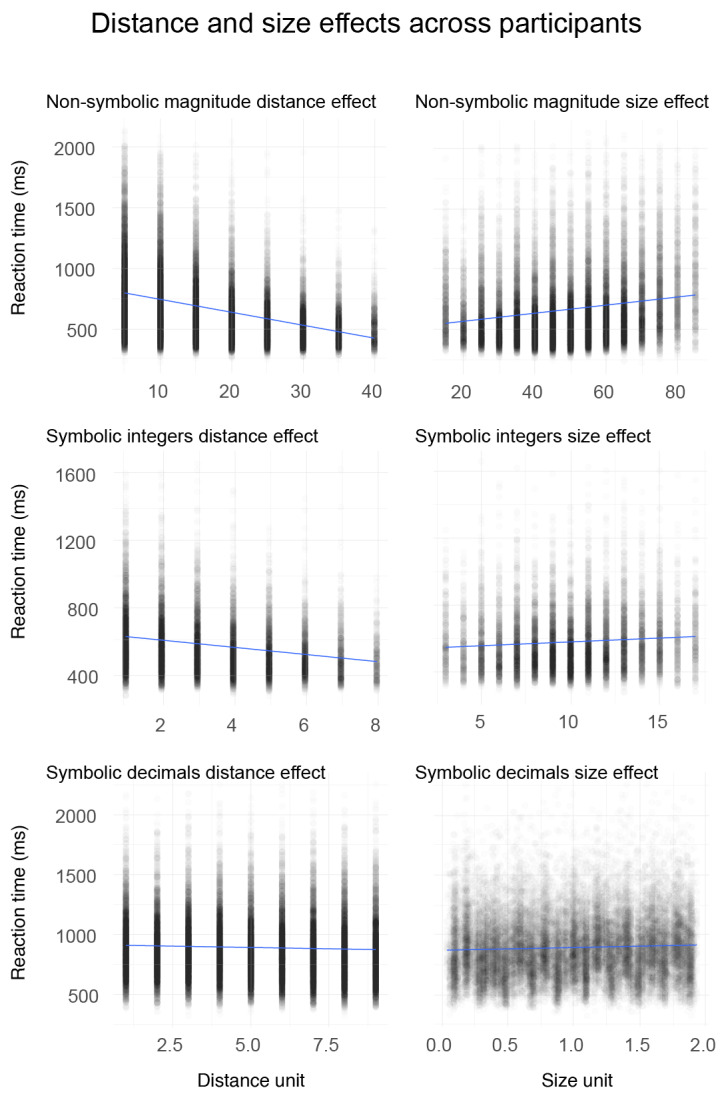
Size (right) and distance (left) effects for non-symbolic magnitude comparison, one-digit integers, and decimals in Experiment 2.

To explore whether the validity of the two theoretical stances varies as a function of inter-individual propensity to demonstrate a significant distance and/or size effect, we computed their correlation in the subsample of participants showing a significant modulation of RT as a function of distance and size (see Figure 4), separately for each task.

**Figure 5:**
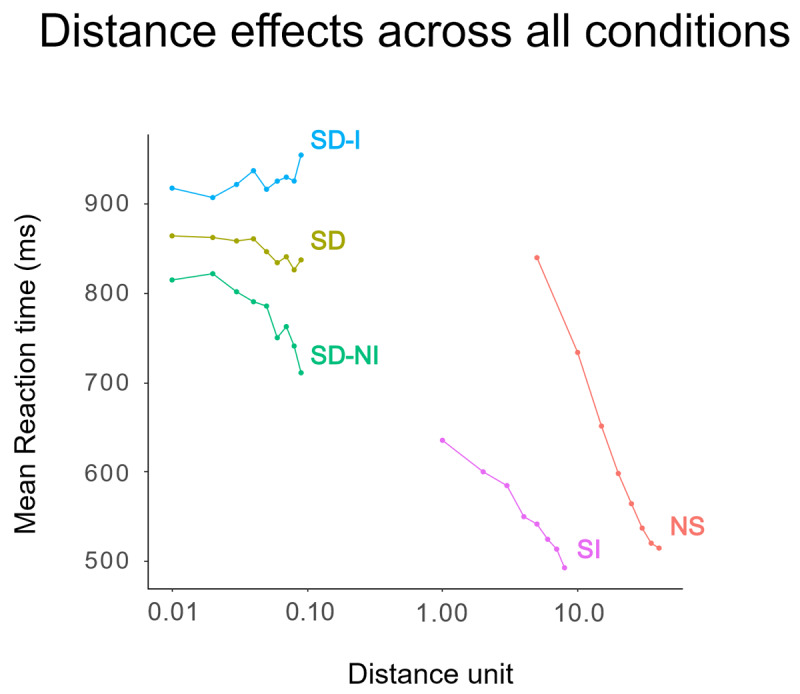
Distance effect for non-symbolic (NS), symbolic integer (SI) and symbolic decimal (SD) conditions, and for symbolic decimal identical DP1 (SD-I) and symbolic decimal non-identical DP1 (SD-NI) sub-conditions in Experiment 2.

All distance (44 participants; 100% of the sample) and almost all size effects (43 participants; 97.73% of the sample) were significant for the non-symbolic format. For symbolic integers, all distance effects were significant but the number of significant size effects dropped by almost half (25 participants; 56.82%). For symbolic decimals, both effects decreased strongly (Distance: 12 participants, 27.27%; Size: 14 participants, 31.82%). A closer look at the decimals revealed that identical DP_1_ had a higher number of distance than size effects that were significant (Distance: 33 participants, 75.00%; Size: 10 participants, 22.73%). The pattern was reversed for non-identical DP_1_ pairs that had more size than distance effects that were significant (Distance: 5 participants, 11.36%; Size: 10 participants, 22.73%).

**Figure 6:**
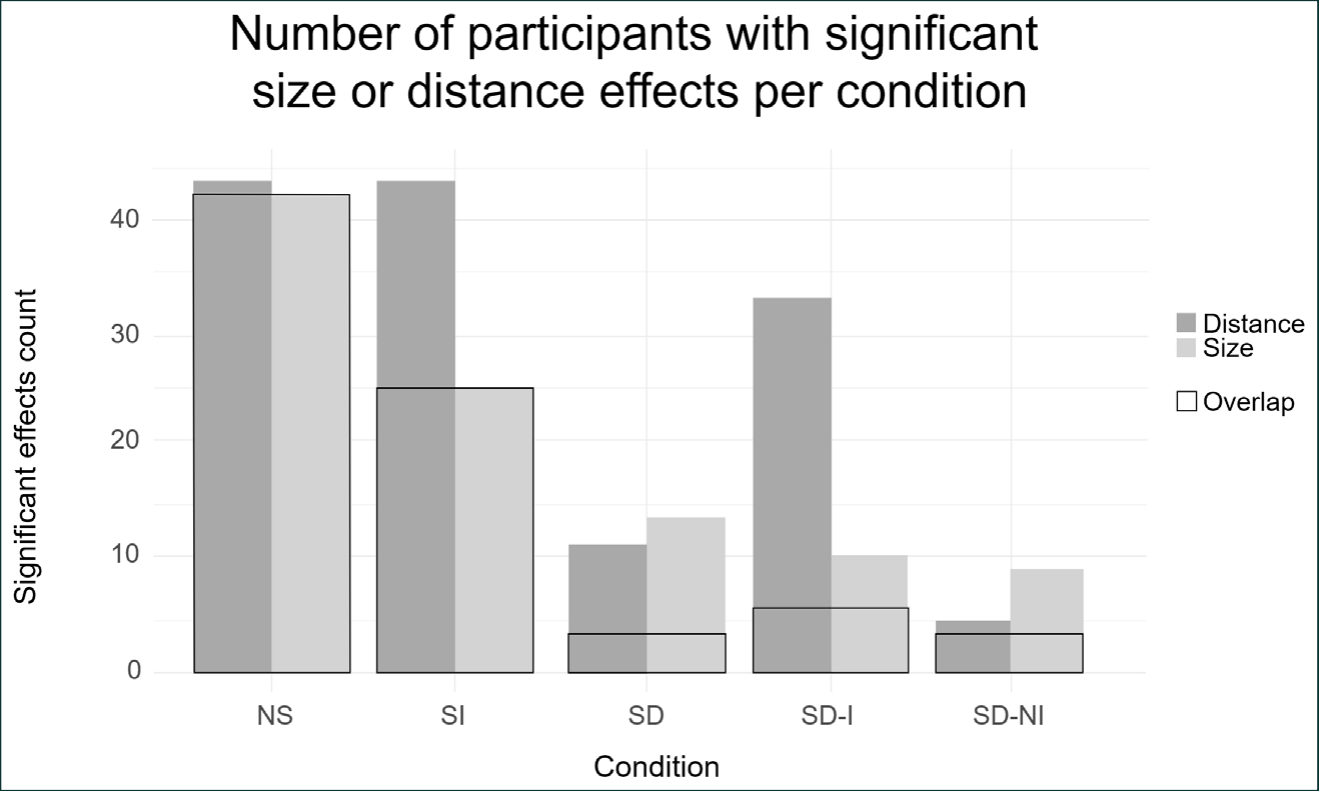
Histogram of participants with significant size or distance effects in Experiment 2 in the dot arrays (NS), one-digit integer (SI), two-digit decimal (SD) conditions, Symbolic decimal identical DP_1_ (SD-I) and Symbolic decimal non-identical DP_1_ (SD-NI) sub-conditions. The black rectangles (overlap) depict the number of participants showing both effects in each condition.

When restricting our analysis to those participants who showed significant size and distance effects at the individual level (n = 43), we obtained a similar correlation coefficient (*r*(41) = –.80; *p* < .001) for the non-symbolic format. For symbolic integers, we excluded 19 participants (n = 25), who did not show significant size and distance effects at the individual level. In contrast to the above analysis, we observed a significant correlation coefficient with a score close to the non-symbolic task (*r*(23) = –.71; *p* < .001). For symbolic decimals, too many participants were excluded by our criteria to allow us to run the correlation analysis (identical DP_1_: n = 6, non-identical DP_1_: n = 3). Only four participants showed both a significant size and distance effect for the Symbolic decimal analysis (see Figure 6).

Our results on the symbolic integer task show a relationship between distance and size effects. Because this task is composed of one-digit integers, the presence of size effects at the individual level cannot be a by-product of a DP_1_ distance effect as in the Symbolic decimal two digits task. However, Size effects are present only in a subset of participants (contrary to the distance effect). This could be due to inter-individual differences in strategies to solve the number comparison tasks. Some individuals may have relied on separate number systems for symbolic and non-symbolic quantities, while others may have relied on a unitary system (i.e. the ANS).

#### Does the task-specific reliability prevent the correlation from emerging?

We computed the split-half reliability and corrected our correlation coefficients for the lack of reliability of our variables following the method described in Krajcsi (2017). This allowed us to correct for the presence of noise in, for example, the visual perception of our stimuli that could differ from one task to another (e.g. dots vs. digits). Different levels of noise could decrease the strength of the correlation between our two effects for a given task, possibly creating artificial differences of correlation coefficients between tasks. We corrected significant correlation coefficients only. The correction increased the strength of our correlation in most cases at the exception of non-identical DP_1_ (Non-symbolic: *r* = –.81; *r*_corrected_ = –.85; Symbolic Integer: *r* = –.19; *r*_corrected_ = –.23; Symbolic decimal identical DP_1_: *r* = .11; *r*_corrected_ = .37; Symbolic decimal non-identical DP_1_: *r* = .44; *r*_corrected_ = .00). It should however be mentioned that the different number of trials from one condition to another should have an impact on their respective reliability scores. Therefore, results must be interpreted with caution.

### Discussion

The results of Experiment 2 by-and-large replicate the findings of Krajcsi (2017). For non-symbolic quantities, we observed a strong and reliable correlation between distance and size effect that was not present in one-digit integers. For less familiar decimals, we found distance and size effects to correlate, potentially driven by the correlation in non-identical DP_1_ (e.g. 0.45–0.58). This could suggest that in situations that require a more in-depth processing of the numerical quantity referred to by number symbols, the ANS may support the decision process. However, this correlation between size and distance effects in non-identical DP_1_ may be misleading, as the size effect in this condition could be a by-product of a distance effect among DP_1_ digits. For decimals with identical DP_1_, we did not observe a correlation of size and distance effect.

Interestingly, we found that in those participants who showed both distance and size effects at the individual level in the one-digit integer task, their strength was highly correlated. This may be taken as a first indication for the idea that the involvement of the ANS in symbolic numerical magnitude comparison tasks shows high variability across participants. Factors that determine this involvement will have to be identified in future studies.

## Experiment 3

To further test the idea that size effects we observed in experiment 2 for positive two-digit decimals reflect canonical distance effects among DP_1_ digits (possibly induced by the number pair comparison paradigm), we decided to reproduce Experiment 1 with decimals as stimuli.

### Participants

We recruited 43 participants via an online platform (https://www.risc.cnrs.fr/), residing in greater Paris region. Participants were compensated with 10 euros for a test duration of one hour. We excluded two participants from the analysis because their error rates were superior to 3 SD above the mean error rate of the group. The final sample included 12 males and 29 females (one participant did not provide this information). The mean age was 25.56 years (ranging from 19 to 34). The experiment was carried out in compliance with the ethical standards of the Declaration of Helsinki and was approved by the local ethics committee (2023-96-KNOPS-GARSMEUR).

### Stimuli and Materials

Similarly, to experiment 1, we used MATLAB to generate stimuli for both formats.

#### Non-symbolic quantities (sets of dots)

Non-symbolic stimuli are identical to those in Experiment 1.

#### Symbolic quantities (two-digit numbers)

We used the same stimulus set as in Experiment 1, but turned them into decimals by adding “0.” in front of each number. Therefore, we presented the numbers 0.11 through 0.99 (except 0.55). Each of the 88 numbers was shown twice.

### Design and Procedure

Design and procedure of Experiment 3 were identical to Experiment 1 with the exception that participants were instructed to compare a number displayed in the center of the screen to the number 0.55 (instead of 55 in Experiment 1) in the symbolic decimal comparison task.

### Analysis

Analyses of Experiment 3 are identical to experiment 1. Applying our exclusion criteria, we excluded two participants. All analyses are based on the data from the remaining 41 participants.

### Results

Accuracy differed between the symbolic and non-symbolic format (Mean = 95.16%, SD = 3.97 and Mean = 62.42%, SD = 4.40, respectively). As in the first experiment, there was a notably higher error rate in the dot condition.

Non-symbolic comparisons (*t*(40) = 6.651; *p* < .001, *d* = 1.05) were slower (Mean (SD) = 695 (188) ms) compared to symbolic two-digits integers (Mean(SD) = 524 (79) ms).

For non-symbolic quantities, linear mixed model on all trials with participants as random intercepts showed a significant effect for distance (*t*(4388) = 3.336, *p* < .001), size (*t*(4388) = 2.144, *p* = .032) but no significant effect for their interaction (*t*(4388) = 0.071, *p* = .943). The second linear mixed model with ratio as the only regressor also yields a significant effect (t(4390) = 19.30, p < .001). For symbolic numerosities, linear mixed model showed significant effect for distance (*t*(6704) = 6.057, *p* < .001), size (*t*(6704) = 2.749, *p* = .006) and their interaction (*t*(6704) = 2.725, *p* = .006). The linear mixed model with ratio as the only regressor also yields a significant effect (*t*(6706) = 12.51, *p* < .001).

Similarly to the first experiment, participants reacted significantly faster to numbers smaller than 0.55 (M = 670 ms, SD = 177 ms) compared to numbers larger than 0.55 (M = 724 ms, SD = 203 ms, *t*(40) = 6.54, *p* < .001, *d* = 0.25) for non-symbolic quantities. Conversely, for the symbolic stimuli, participants reacted faster to numbers larger than 0.55 (M = 519 ms, SD = 82 ms) compared to numbers smaller than 0.55 (M = 528 ms, SD = 79 ms, *t*(32) = 2.51, *p* = .016, *d* = 0.11).

For non-symbolic quantities, the distance effect as measured via the slope of the regression of number on RT quantities was larger for numbers smaller than 0.55 (M = 6.80 ms/number, SD = 4.03 ms) compared to quantities larger than 0.55 (M = 4.92 ms/number, SD = 4.57 ms, *t*(32) = 2.77, *p* = .008, *d* = 0.46). Similarly, for symbolic stimuli, the distance effect was more pronounced for numbers smaller than 0.55 (M = 304 ms/number, SD = 181 ms) compared to numbers larger than 0.55 (M = 181 ms/number, SD = 154 ms; *t*(40) = 5.28, *p* < .001, d = 0.75). These results closely follow the results of the first experiment. It is important to note that the means and standard deviation of the symbolic condition are two orders of magnitude superior to the ones of the first experiment. This difference is due to the fact that regressions were calculated on the same number as the first experiment divided by one hundred. The fact that reaction times were not adjusted to the current stimulus sets (by being the same order of magnitude as in the first experiment) suggests that participants may have ignored the leading zero and accessed the same underlying representation in both tasks (two-digit integers and decimals).

The estimated correlation coefficient for the non-symbolic task was *r*(39) = –.32 (*p* = .041), a more moderate degree of correspondence between size and distance than in the previous experiments, albeit still significant. For the symbolic task the estimated correlation coefficient was not significant (*r*(39) = .14; *p* = .392). There was an overlap between confidence intervals of correlation coefficients of both non-symbolic (95% CI[–.61, –.03]) and symbolic tasks (95% CI[–.17, .45]). Therefore, we can’t exclude with at least 95 percent confidence that our results are due to random sampling. We also computed the correlation between the ratio effects of both formats (*r*(39) = 0.26, *p* = .099).

As in the first experiment, we re-ran our analyses only with those participants who show both significant distance and size effects at the individual level. In the non-symbolic task, most distance effects (32 participants; 78.05% of the sample) and a small portion of size effects (15 participants; 36.59% of the sample) were significant. For symbolic two-digit integers, most of the distance effects were significant (34 participants; 82.93% of the sample), but the number of significant size effects was greatly reduced (4 participants; 9.76%). As in the first experiment, in the case of the symbolic two-digit integers the low number of remaining participants prevented us from running a meaningful regression analysis. In the non-symbolic task, the correlation between significant size and distance effects was no longer significant (n = 14, *r*(12) = –.44, *p* = .119).

The split half reliability analysis correction increased the strength of our correlation in both format conditions (Non-symbolic: *r* = –.32; *r*_corrected_ = –.53; Symbolic two digits integer: *r* = .14; *r*_corrected_ = .23).

### Discussion

Using the same paradigm as in the first experiment but with the stimulus set of the second experiment allowed us to disentangle between the effects of paradigm and stimulus sets on participants’ response latencies. In Experiment 2, the correlation between the size and distance effects that we found among non-identical DP_1_ numbers seems to reflect a correlation between a distance effect among DP_1_ digits and a distance effect among DP_2_ digits. Therefore, we reproduced Krajcsi’s (2017) findings using two-digit decimals.

It is important to notice that the use of a fixed reference paradigm substantially affected the pattern of results. While we observed componential distance effects (i.e. distance effects that reflected DP_1_ or DP_2_ distances) in experiment 2, reaction times in Experiment 3 reflect mainly overall distance between the current number and the fixed reference. This suggests that participants relied to a larger extent on the overall magnitude rather than engaging in componential comparison of the constituting digits of a given number.

Thus, when presenting two to-be-compared numbers simultaneously, people rely on distinct representations of limited magnitude range instead of one unitary representation of the overall magnitude of a given number. In this context participants engage in digit-wise comparison strategies rather than comparing overall magnitude (Knops, 2006). This divergence from one paradigm to another is in line with previous findings (Dehaene et al. 1990, Nuerk et al., 2001).

## General Discussion

This study set out to test the idea that a unitary analog magnitude representation underlies both symbolic and non-symbolic magnitude comparison against an alternative view that assumes that distance and size effects are driven by different factors in both formats. In line with the latter, previous studies (e.g. Krajcsi, 2017) showed that size and distance effects only correlate in non-symbolic magnitude comparisons, but not in tasks using symbolic stimuli (i.e. Arabic digits). To make this general claim, we need to exclude the possibility that these results were due to the overlearned nature and the limited numerical range that was used in Krajcsi (2017). We ran three experiments in which we correlated size and the distance effects in magnitude comparison tasks using symbolic stimuli with a larger numerical range (two-digit numbers in experiment 1) or less familiar numbers (decimals in Experiments 2 and 3). In all experiments, we additionally replicated Krajcsi (2017) by including a non-symbolic numerosity comparison task. Going beyond the original study in which numerosity was systematically and uniformly correlated with non-numerical features (i.e. overall occupied area and convex hull), we decorrelated numerical from non-numerical features to increase participants’ reliance on the numerical stimulus dimension rather than non-numerical features.

Overall, we replicated the results reported by Krajcsi (2017). In all experiments, we observed a significant correlation between size and distance effect for non-symbolic numerosity comparison. In contrast, our in-depth analyses of the performance patterns in symbolic numbers allowed us to exclude the presence of a significant correlation between generic size and distance effects. This was true for single-digit integers, two-digit integers and decimals. Together, these results corroborate the idea that symbolic number processing relies on number representations that are partially distinct from the ANS. This dovetails with the view that exact number skills and mathematics have emerged via cultural practices governed by language-driven symbolic reasoning (Nunez, 2017). In particular, these results are in line with the idea that size and distance effects are driven by distinct sources in symbolic number formats, objecting the nativist view that symbolic and exact mathematical capacities originate from an innate core numerical abilities (e.g. Dehaene, 2008).

This overall interpretation needs to be qualified since this pattern was not identical for all stimulus classes. In particular, when analyzing decimals overall (i.e. with identical and non-identical DP_1_) and decimals with non-identical DP_1_, we observed a significant correlation between the two effects that could be interpreted as an involvement of a unitary system for this range of stimuli. We are inclined to refute this interpretation based on several strands of evidence. First, the correlation was significantly smaller compared to the correlations observed in non-symbolic formats. Second, size was positively correlated with distance rather than negatively. Third, the LMM revealed no sign of an interaction between distance and sum of the to-be-compared numbers, suggesting the presence of two distance effects that operate among DP_1_ and DP_2_ digits respectively. Finally, Experiment 3 failed to replicate the correlation between size and distance using a fixed reference paradigm, underlining the idea that in experiment 2, participants engaged in a componential comparison of the constituting digits rather than a comparison of the overall magnitude that would be necessary to conclude that a unitary magnitude system drives performance.

In light of these results, it seems that the presence of a higher proportion of “size effects” among non-identical DP_1_ comparisons in Experiment 2 is not indicative of an actual size effect per se, but is a by-product of participants’ attention to DP_1_ digits and therefore corresponds to a distance effect between DP_1_ numbers. This DP_1_ distance effect was higher in proportion among non-identical DP_1_ pairs, as these could be solved by solely focusing on the DP_1_ digit.

Conversely, the proportion of participants showing significant distance effects was higher than the proportion of participants with significant size effects for identical DP_1_ pairs where attending to the unit digit was sufficient to give correct answers and paying attention to the decade number was irrelevant.

One reason that prevents us from rejecting the idea of a unitary system underlying numerical magnitude comparison altogether is the observation that size and distance effects not only varied in our samples but they significantly correlated in those participants who – at the individual level – demonstrated both effects. From a purely psychometric point of view, it is then not surprising that we failed to observe a correlation between two effects in the entire sample if these effects are not present at the individual level in the first place. At a more theoretical level, these results may imply that the mechanisms recruited in a magnitude comparison task vary between participants. Restricting the correlation analysis to those who do manifest both effects at the individual level is not a conservative way of identifying those who rely on the ANS. It does not – in and by itself – predict that there is a significant correlation between these effects. Therefore, the presence of such correlation among a subset of participants is not trivial and might be indicative that while some individuals rely on a unitary ANS, others may have developed a parallel DSS that they rely on in symbolic number comparisons. Most participants belonged to the second category; therefore, we acknowledge that the majority of participants did not rely on an ANS-type of processing in the symbolic condition. Factors that determine these mechanisms will have to be identified in future studies.

Alternatively, one may argue that the numerical magnitude comparison task is not suited for revealing the size effect in the symbolic domain. Under this assumption, the size effect would have been present and a common magnitude system would govern numerical quantity processing across different formats. The absence of the correlation between size and distance effect would be mainly due to a measurement problem: the numerical magnitude comparison with symbolic numbers would not allow the size effect to be evaluated.

We chose a widely used numerical magnitude comparison task to assess the processes that govern the semantic processing of numerical information. We changed the numerical range of the stimuli in the symbolic number comparison to allow for a larger variability of reaction times due to lowered familiarity. The size effect may have remained elusive despite this change and future studies may adopt a larger stimulus range or different paradigms to probe the size effect. It should be noted that the size effect is indeed one of the hall-mark effects that is assumed at the theoretical level but surprisingly few studies have assessed it in the symbolic format. Against this background we think our results represent an important contribution by demonstrating that the size effect remains elusive and that future studies would need to explore the reasons for this discrepancy between the standard theoretical model (i.e. logarithmically compressed number line) and the empirical findings.

The ANS account relies on the idea that a holistic magnitude code determines performance (Dehaene, 2008). In contrast, a rich body of evidence suggests that multi-digit numbers are processed in a hybrid fashion. Beyond the holistic magnitude code, hybrid models incorporate distinct magnitude codes of the digits that constitute a given numeral (Nuerk et al., 2001; Nuerk et al., 2014). Since the emergence of a size effect critically hinges on the holistic, overall magnitude code, current experiments were designed from the ANS point of view. The absence of the size effect in many participants may be interpreted as the consequence of a decomposed processing that represents an additional noise source and may dilute the impact of the holistic magnitude code and even dominate the magnitude processing. The reverse distance effect in Experiment 2 can be interpreted as evidence for this assumption. Performance in Experiments 1 and 3, however, was strongly modulated by overall distance (cf. Figure 1). Even in this context, the size effect remained elusive. The current data do not allow us to disentangle the impact of decomposed processing or the prevalence of decomposed over holistic processing strategies in more detail. Future studies could address this issue, for example by varying the percentage of within-decade trials (e.g. 41–46) in two-digit number comparisons and exploring the potential modulation of the correlation between size and distance effect.

Our results show that more attention should be given to inter-individual differences, in particular concerning the presence of size effects in the context of paradigms probing the symbolic representation of quantities. Participants that showed a size effect in the one-digit integer task seemed to use the ANS to solve the task, as suggested by the high correlation between the two effects. For the remaining participants, we can only speculate that either a size effect is present that we did not succeed to measure or no size effect is present and participants rely on different mechanisms to solve symbolic and non-symbolic tasks. The significant difference between distance effects before and after the reference number in Experiments 1 and 3 suggests that we did not succeed to capture the size effect with classical measures. Alternatively, the cognitive mechanisms used to solve the symbolic tasks may be deprived of any size effect contrary to the predictions of the DSS account.

To conclude, our results do not support the idea that a unitary magnitude system (e.g. the approximate number system) governs numerical magnitude comparison performance in symbolic formats even if the particular number range is less familiar (i.e. decimals; two-digit numbers). This notion does, however, not hold for all participants alike. In particular, participants who exhibit significant size and distance effects at the individual level, show a pattern of performance that is consistent with the unitary system view. Previous neuroimaging studies testing the overlap of symbolic and non-symbolic representations using multivariate pattern analysis of fMRI data have found similar results relating to inter-individual differences in the presence of distance and size effects (Wilkey et al., 2020). Furthermore, there is an inverse correlation between neural overlap and participants’ arithmetic fluency. Individuals with high correlation between symbolic and non-symbolic representations exhibit poorer performance in arithmetic (Bulthé et al., 2018). This suggests that a more efficient mapping was achieved as neural overlap dissociated, which is an argument in favor of symbolic estrangement (Lyons et al., 2012). Future studies need to describe the principles that shape these interindividual differences and their relation to symbol grounding.

## Limitations

The current series of experiments assesses the correlations between size and distance effects in two numerical magnitude comparisons in symbolic and non-symbolic format. On a more general note, we acknowledge that correlations between two measures may, in some cases, reflect the influence of a third, unmeasured factor that drives both measures simultaneously. Prominent candidates for such overarching influences include processing speed or general intelligence, which are known to affect a wide range of cognitive tasks. Alternatively, because the present experiments primarily focus on comparing correlations observed in symbolic versus non-symbolic formats, it is important to keep in mind that unmeasured factors may differentially impact format-specific processes. For instance, the need to inhibit non-numerical stimulus features is a key demand in non-symbolic numerosity comparison, whereas such interference does not arise to the same extent in symbolic number comparison. In the current series of experiments, we did not assess any external factors and hence cannot exclude that our results reflect their impact. Assessing and systematically exploring these external factors represents an interesting avenue for future experiments.

In Experiments 1 and 3, we adopted a holistic processing framework. Numerical distance was defined as the absolute difference between each target and the reference value (e.g. |32–55| = 23). Although digit-by-digit comparisons cannot be ruled out entirely, evidence from Experiment 2 and previous work (Nuerk et al., 2001) supports hybrid processing. It should be noted, though, that overall distance represents a strong predictor for performance in Experiments 1 and 3. Therefore, our results may not be reproducible under a decompositional model assumption in which distance and size effects are computed by separate mechanisms.

It should also be noted that the split-half reliability of the distance effect in our data was comparable to the reliabilities observed by Krajcsi (2017; .56 and –.19 for Experiments 1 and 2, respectively; personal communication). Reliabilities of the size effect, however, were lower than in Krajcsi (2017, .61 & .49 for Experiments 1 and 2, respectively; personal communication). This is potentially due to the low proportion of significant size effects in our data. If there is no significant size effect for a participant, splitting the dataset in half and computing the size effects for both ensembles is not expected to give a significant correlation between the two. Therefore, we argue that the low reliability in this case, like the low proportion of significant effects from which it derives, can be understood as a consequence of the variability of strategies used to solve the task. The smaller number of trials in the symbolic compared to the non-symbolic format may have lowered the reliability and hence negatively impacted the emergence of a correlation between size and distance effect in these experiments. Despite these considerations, our results must be interpreted with caution. Both the overall low reliability or a genuine absence of correlation may account for the current findings.

From one experiment to another, a number of design features differ (e.g. interstimulus duration, the number of stimuli in each condition, total experiment duration, response button etc). In addition to differences in paradigms (reference number vs. number pair comparison), these design parameters may have contributed to the differences observed from one experiment to another (in particular between experiment 2 and the other).

## Data Accessibility Statement

All supplementary materials, raw data and analysis scripts, are publicly available at: https://osf.io/7y38h/?view_only=d6edb4df583742ee8de27c22dac045cb.
